# 1256. Changes in Antimicrobial Stewardship (AS) Core Element Implementation among Oregon Hospitals following the COVID-19 Pandemic

**DOI:** 10.1093/ofid/ofad500.1096

**Published:** 2023-11-27

**Authors:** Jessina C McGregor, Lisa Iguchi, Elizabeth Breitenstein

**Affiliations:** Oregon State University, Portland, OR; Oregon Health Authority, Portland, Oregon; Oregon Health Authority, Portland, Oregon

## Abstract

**Background:**

The CDC established core elements for effective hospital AS programs, which includes a team with dedicated effort and appropriate experience. CDC further identified that AS leadership with infectious disease specialty training enhances quality and impact of AS. Since the COVID-19 pandemic, facilities have reported widespread challenges to maintaining healthcare worker staffing. Our objective was to compare AS core element implementation in Oregon hospitals before and after the pandemic.

**Methods:**

We performed a secondary analysis of 2019 and 2022 NHSN annual facility survey data from acute care and critical access hospitals in the state of Oregon. The frequency of implementation of core elements was compared between time frames using the chi-square test. Secondary analyses were performed among acute care and critical access hospitals.

**Results:**

Overall, 59 of 60 Oregon hospitals responded to the 2019 facility survey and 58 to the 2022 survey. In 2019, 76% reported having implemented all core elements compared to 95% in 2022 (*p* < 0.01; Figure 1). Among acute care hospitals, implementation of all elements was 77% (27/35) in 2019 and 100% (33/33) in 2022; among critical access hospitals, it was 75% (18/24) and 88% (22/25) respectively. Significantly more hospitals implemented the education core element in 2022 (100%) compared to 2019 (80%; *p* < 0.01). Hospitals, overall, had a decreased frequency of physician/pharmacist co-led AS programs in 2019 vs. 2022 (75% vs 66%, *p* = 0.28). Decreases of co-led programs were mainly observed in critical access hospitals (71% vs. 44%; *p* = 0.06), where both pharmacist (21% vs. 32%; *p* = 0.38) or physician-led programs (4% vs. 13%; *p* = 0.32) increased. The proportion of pharmacist leads/co-leads with infectious diseases specialty training also increased non-significantly (29% vs 36%, *p* = 0.39; Figure 2).

**Figure 1.**
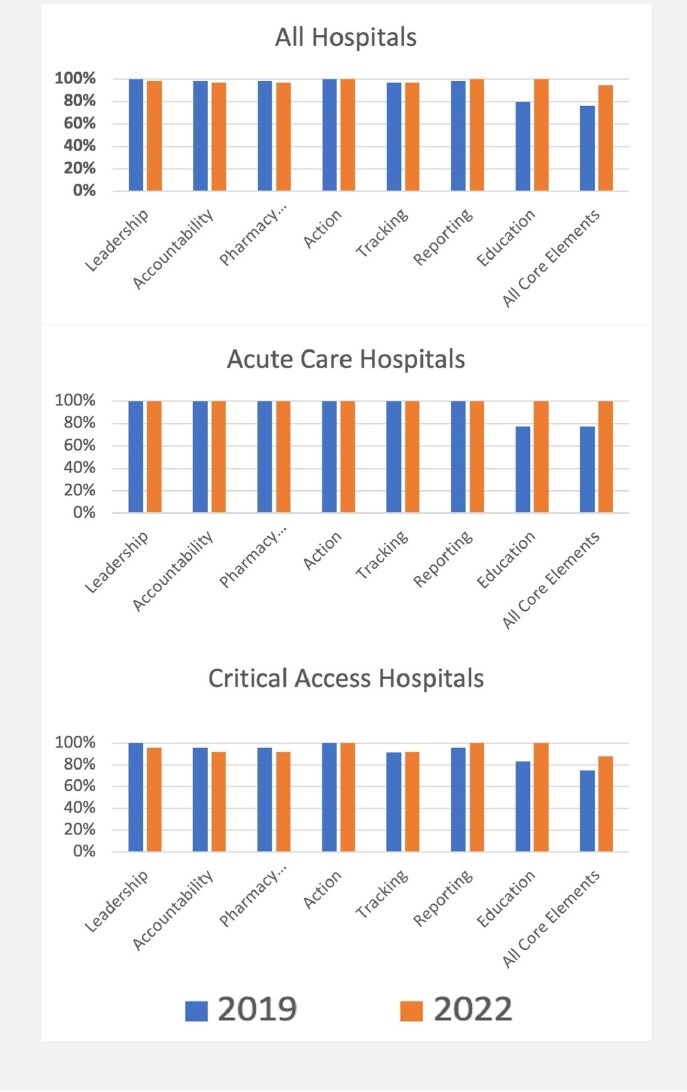
Implementation of Antimicrobial Stewardship Core Elements in Oregon Hospitals

**Figure 2.**
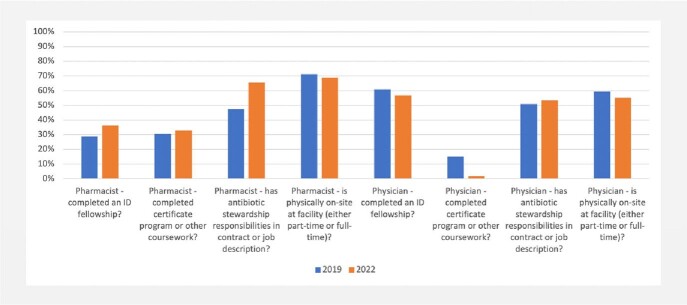
Characteristics of Antimicrobial Stewardship Leadership

**Conclusion:**

Despite challenges stemming from the COVID-19 pandemic, hospital commitment to antimicrobial stewardship increased as indicated by greater implementation of the core elements. Yet gaps persist for critical access hospitals; further efforts are needed to support to full implementation in this setting.

**Disclosures:**

**All Authors**: No reported disclosures

